# *Lactobacillus delbrueckii* subsp. *lactis* CKDB001 Ameliorates Metabolic Complications in High-Fat Diet-Induced Obese Mice

**DOI:** 10.3390/nu16244260

**Published:** 2024-12-10

**Authors:** Hyunsoo Jang, Hyunchae Joung, Jaeryang Chu, Minseo Cho, Yeon-Woo Kim, Kyung Hwan Kim, Chang Hun Shin, Jisu Lee, Jung-Heun Ha

**Affiliations:** 1 Department of Food Science and Nutrition, Dankook University, Cheonan 31116, Republic of Korea; 2 Microbiome Research Laboratory, Chong Kun Dang Bio (CKDBiO) Research Institute, Ansan 15604, Republic of Korea; 3 Chong Kun Dang Bio (CKDBiO) Research Institute, Ansan 15604, Republic of Korea; 4 Research Center for Industrialization of Natural Neutralization, Dankook University, Yongin 16890, Republic of Korea

**Keywords:** MASLD, probiotics, *Lactobacillus delbrueckii* subsp. *lactis* CKDB001

## Abstract

Background/Objectives: Functional probiotics, particularly *Lactobacillus delbrueckii* subsp. *lactis* CKDB001, have shown potential as a therapeutic option for metabolic dysfunction-associated steatotic liver disease (MASLD). However, their effects have not been confirmed in in vivo systems. Here, we investigated the effects of *L. delbrueckii* subsp. *lactis* CKDB001 on insulin resistance, dyslipidemia, MASLD, and lipid metabolism in a murine model of high-fat diet (HFD)-induced obesity. Methods: The mice were divided into four groups (*n* = 12 per group)—normal chow diet (NCD), high fat diet (HFD), HFD with *L. delbrueckii* subsp. *lactis* CKDB001 (LL), and HFD with resmetirom (positive control (PC), a thyroid receptor β agonist). The experimental animals were fed NCD or HFD for 12 weeks, followed by an additional 12-week oral treatment with LL or resmetirom. Results: LL supplementation reduced body weight, insulin levels, and HOMA-IR compared with those in the HFD group, indicating improved insulin sensitivity. Additionally, LL reduced serum triglyceride (TG) levels without affecting total cholesterol (TC) levels. HFD consumption increased liver weight and hepatic TG and TC levels, indicating ectopic fat accumulation; however, LL supplementation reversed these changes, indicating a liver-specific effect on cholesterol metabolism. Furthermore, LL administration attenuated NAFLD activity scores, reduced hepatic fibrosis, improved liver function markers (aspartate aminotransferase), and enhanced Adenosine monophosphate-activated protein kinase (AMPK) phosphorylation. However, LL did not considerably affect the expression of genes related to lipid metabolism. In epididymal adipose tissue, LL treatment reduced leptin levels but had no effect on adiponectin; additionally, histological analysis showed an increase in adipocyte size, potentially linked to enhanced energy metabolism. Conclusions: Collectively, these findings suggest that LL could be a promising therapeutic candidate for improving insulin sensitivity, reducing hepatic lipid accumulation, and mitigating MASLD.

## 1. Introduction

Obesity, a condition characterized by the accumulation of excess white adipose tissue (WAT), resulting in a body mass index (BMI) ≥ 30 kg/m^2^, has become a global epidemic with major implications for public health [1]. Over the past few decades, the prevalence of obesity has considerably increased, driven by factors such as sedentary lifestyles [2] and the widespread adoption of Western dietary patterns [3], particularly high-fat diets (HFDs). According to the World Health Organization (WHO), the number of individuals with obesity has nearly tripled since 1975, with current estimates suggesting that approximately 1.3 billion adults worldwide are overweight, and more than 600 million of them are obese [4]. Obesity is not only an issue of excess weight but also a major risk factor for several chronic diseases. Excessive body fat accumulation increases the risk of developing various complications, including cardiovascular diseases (CVD) [5], type 2 diabetes (T2D) [6], hypertension [7], and cancer [8,9]. To address these worsening health outcomes, analyzing their underlying mechanisms and developing effective preventive strategies to mitigate the impact of obesity on overall physiological homeostasis is crucial.

One such remarkable impact is the negative effect of obesity on hepatic homeostasis. Obesity is a major risk factor for the development of non-alcoholic fatty liver disease (NAFLD), a condition characterized by excessive fat accumulation in the liver [10]. NAFLD occurs when ectopic fat builds up in the liver without the influence of alcohol consumption and may progress to more severe pathologic conditions such as hepatitis, fibrosis, and cirrhosis [11]. Recently, NAFLD was renamed as metabolic-associated fatty liver disease (MASLD) to emphasize the strong connection to metabolic abnormalities beyond just fat accumulation independent of alcohol consumption [12]. MASLD is estimated to affect approximately 30% of the global adult population, and the 15% increase in MASLD prevalence between 1991 and 2019 corresponds with the rise in obesity and obesity-related diseases [13,14]. The condition elevates the risk of developing hepatomegaly, excessive ectopic WAT accumulation in the liver, insulin resistance, dyslipidemia, and reduced lipid metabolism [15,16,17].

Currently, lifestyle changes involving dietary regulation and exercise are the primary preventive and therapeutic options for mitigating the MASLD caused by obesity. Although various therapeutic drugs have been released in the clinical market—such as pioglitazone, which improves insulin sensitivity through PPARγ activation [18,19]; GLP-1 receptor agonists such as liraglutide, which aid in weight loss by modulating appetite [20,21]; and SGLT2 inhibitors such as empagliflozin, which can lower blood glucose [22,23]—these medications are often associated with several side effects. For instance, pioglitazone can cause weight gain, fluid retention, and increased risk of heart failure [18,19]; liraglutide may cause nausea and vomiting; and empagliflozin may increase the risk of urinary tract infections and dehydration [21]. Given the challenges in developing effective pharmaceutical treatments, it is necessary to explore natural materials with beneficial effects and few side effects. However, medications alone cannot serve as preventive measures against obesity-induced MASLD; therefore, preventive approaches such as consuming functional nutraceuticals and probiotics are necessary.

Probiotics are defined by the Food and Agriculture Organization and WHO as “live microorganisms that, when administered in adequate amounts, confer a health benefit on the host” [24]. Healthy gut microbiota are essential for proper metabolic functions and homeostasis, playing a crucial role in the catabolism and anabolism of energy derived from diet [25,26]. Accordingly, the use of probiotics to selectively regulate the gut microbiota has emerged as a potential treatment strategy for managing obesity. Probiotics reduce WAT mass and positively regulate metabolic homeostasis [27]. For instance, studies on murine models of obesity induced by an HFD have demonstrated that probiotic treatment can alter bile acid composition and kinetics, thereby ameliorating insulin resistance [28]. Additionally, probiotic treatment has been found to alleviate dysbiosis caused by a high-fat diet by reducing total cholesterol (TC), triglycerides (TG), and LDL-C levels, as well as mitigating dyslipidemia [29]. While numerous studies have documented the effects of probiotics on obesity and related disorders, research on using probiotics to specifically modulate obesity and its associated metabolic complications remains at an early stage.

Among probiotics, the genus *Lactobacillus*, comprising Gram-positive, thermophilic bacteria, is particularly noteworthy for its economic importance in industrial applications. *Lactobacillus* species are notable for their ability to inhibit the growth of competing microorganisms by depleting available carbon sources and rapidly fermenting organic acids as metabolites [30]. Studies have shown that certain *Lactobacillus* strains are health-promoting and exhibit low levels of antibiotic resistance; thus, they are widely used as probiotic treatments in animal models of obesity. Among the 42 species within *Lactobacillus*, *L. delbrueckii* subsp. *lactis* is particularly important for the commercial production of fermented products such as yogurt and cheese [31]. Previous studies have demonstrated that *L. delbrueckii* subsp. *lactis* can reduce fasting blood glucose levels, inhibit the growth of pathogenic bacteria, and ameliorate ulcerative colitis [32,33,34]. Furthermore, we recently demonstrated the safety and effectiveness of *L. delbrueckii* subsp. *lactis* CKDB001 as a probiotic and potential therapeutic candidate for MASLD [35]. *L. delbrueckii* subsp. *lactis* CKDB001 reduces lipid accumulation in hepatic cells by promoting the expression of genes involved in fatty acid oxidation and suppressing that of those involved in fatty acid uptake [36]. Given its crucial role in reducing lipid accumulation in vitro, we aimed to evaluate the impact of *L. delbrueckii* subsp. *lactis* CKDB001 in vivo, which remains largely unexplored. Specifically, we examined the influence of *L. delbrueckii* subsp. *lactis* CKDB001 on insulin resistance, dyslipidemia, MASLD, and lipid metabolism in an HFD-induced obese murine experimental model.

## 2. Materials and Methods

### 2.1. Preparation for Probiotic Powder

*Lactobacillus delbrueckii* subsp. *lactis* CKDB001 (*L. lactis* CKDB001, KCTC 14149BP) was isolated from fermented milk. The *L. lactis* CKDB001 was inoculated into a flask containing de Man, Rogosa, and Sharpe (MRS) broth (BD/Difco, Franklin Lakes, NJ, USA) and incubated under facultative anaerobic conditions at 37 °C for 24 h. Stock solutions were prepared by mixing the cultured MRS broth with an equal volume of a 20% glycerol solution and then stored at −80 °C until use.

The seed culture was grown in a flask with MRS broth at 37 °C for 24 h. The cultured broth was subsequently inoculated into an optimized medium in a mini jar fermenter (Bio Control & Science, MARADO-05D-PS, Xiamen, Fujian, China). Fermentation was conducted at a constant pH of 5.5 to 6.0, maintained automatically by adding NaOH solution (25% *w*/*v*), with agitation at 120 rpm at 37 °C for 16 to 18 h. At the end of fermentation, cells were harvested by centrifugation at 6000 rpm for 10 min (Hanil, Supra R12, Daejeon, Republic of Korea). The resulting cell concentrate (40×) was lyophilized following the manufacturer’s instructions (Cooling & Heating System, Lab-Mast 10, Seoul, Republic of Korea). After lyophilization, the colony-forming units (CFU) per gram of probiotic powder were determined using a serial dilution method. For daily use, *L. lactis* CKDB001 was suspended in phosphate-buffered saline (PBS, Bioneer, Daejeon, Republic of Korea) and adjusted to a concentration of 1.0 × 10^9^ CFU per mouse.

### 2.2. Animal Experiments and Dietary Interventions

**Figure 1:**
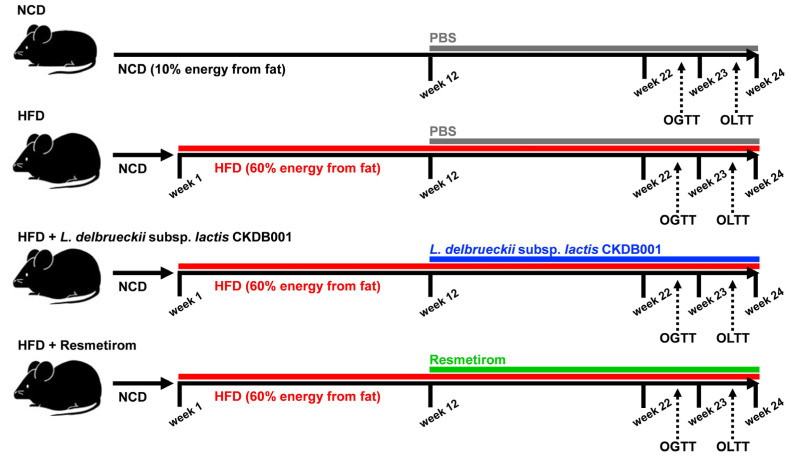
Overview of experimental design. Four-week-old male C57BL/6J mice (*n* = 12 per group) were subjected to the following dietary interventions for 24 weeks: normal chow diet (NCD), high-fat diet (HFD), HFD with *L. delbrueckii* subsp. *lactis* CKDB001 (LL), and HFD with resmetirom (positive control). Abbreviation: HFD, High-fat diet; LL, *L. delbrueckii* subsp. *lactis* CKDB001; NCD, normal chow diet; OGTT, Oral glucose tolerance test; OLTT, Oral lipid tolerance test; PBS, Phosphate buffered saline.

Four-week-old male C57BL/6J mice were purchased from DooYeol Biotech (Seoul, Republic of Korea) and housed under controlled conditions with a temperature of 20 ± 2 °C, humidity of 55 ± 5%, and an alternating 12 h light/dark cycle. After a 1-week acclimation period, the mice were divided into the following four groups (*n* = 12 per group): NCD (normal chow diet, based on the AIN-93G formulation; 10% of energy from fat), HFD (60% of energy from fat; Research Diets Inc., New Brunswick, NJ, USA), LL (HFD with *L. delbrueckii* subsp. *lactis* CKDB001), and PC (positive control, HFD with resmetirom). The experimental animals were fed either an NCD or HFD for 12 weeks. All the HFD-fed groups were provided with drinking water containing fructose at a concentration of 12.5%. After feeding an NCD or HFD to each group for 12 weeks, *L. delbrueckii* subsp. *lactis* CKDB001 or resmetirom was additionally administered orally for another 12 weeks. The LL group mice received *L. delbrueckii* subsp. *lactis* CKDB001 in PBS containing probiotic strains at 1.0 × 10^9^ CFU/g once daily for 12 weeks. The PC group was treated with resmetirom (MedChemExpress, Beytelsbach, Germany) at a dosage of 3 mg/kg (Figure 1). The resmetirom dose was selected based on previous studies [37,38,39]. The NCD and HFD groups were administered the same amount of PBS instead of the treatments. Throughout the experiment, water and food were provided ad libitum. After a 12 h fasting period, the mice were sacrificed via thoracotomy after CO_2_ narcosis. Whole blood samples were collected via cardiac puncture and centrifuged at 3000× *g* and 4 °C for 20 min to obtain the serum. The liver, epididymal adipose tissue (EAT), subcutaneous adipose tissue (SAT), and mesenteric adipose tissue (MAT) were immediately weighed and stored at −80 °C until further analysis. All murine experimental procedures were approved by the Institutional Animal Care and Use Committee of Dankook University (No. DKU 2023-22-085).

### 2.3. Oral Glucose Tolerance Test (OGTT) and Oral Lipid Tolerance Test (OLTT)

Following 22 weeks of dietary intervention, an OGTT was conducted on the mice after a 12 h fast. Briefly, a glucose solution (MB Cell, Seoul, Republic of Korea) was orally administered at a dose of 1 g/kg, and blood samples were collected from the tail vein 30, 60, 90, 120, and 240 min after glucose administration. Thereafter, blood glucose levels were measured using an Accu-Chek instant blood glucose meter (Accu-Chek, Seoul, Republic of Korea).

An OLTT was conducted on the mice after 12 h of fasting following 23 weeks of dietary intervention. Briefly, the mice were orally administered olive oil (Sigma-Aldrich, St. Louis, MO, USA) at a dose of 5 mL/kg, after which blood samples were collected from the tail vein 30, 60, 90, 120, and 240 min post-administration. Blood lipid levels were analyzed using Barogen triglyceride test strips and a Barogen lipid meter (Handok, Anseong, Republic of Korea) [40].

### 2.4. Analysis of Metabolic Parameters in the Serum

Hepatic function was evaluated by quantifying the serum levels of alanine aminotransferase (ALT) and aspartate aminotransferase (AST) using commercial kits (ALT assay, AST assay kit; Embiel, Gunpo, Republic of Korea). Additionally, the serum levels of triglycerides (TGs; Asan, Hwaseong, Republic of Korea), TC (Asan), HDL-C (Asan), and glucose (Crystal Chem, Downers Grove, IL, USA) were measured using commercial kits following established protocols. Low-density lipoprotein cholesterol (LDL-C) [41,42] and cardiac risk factor (CRF) calculations [6] were performed based on prior research.

### 2.5. Histopathological Evaluation of the Liver and EAT

Liver and EAT samples were fixed using 10% formalin and embedded in paraffin. Thereafter, sections were prepared for analysis using hematoxylin and eosin (H&E) and sirius red staining. Fatty liver classification followed the non-alcoholic steatohepatitis (NASH) clinical research network scoring system for NAFLD, with grades ranging from 0 to 3 based on the extent of steatosis [43]. Inflammation was graded from 0 to 3 based on foci per field. The NAFLD activity score (NAS) was calculated based on Kleiner’s criteria, which consider steatosis, lobular inflammation, and ballooning. Patients were grouped into the following NAS categories for statistical analysis: NAS 0–2 (probable no NASH), NAS 3–4 (borderline), and NAS 5–8 (probable NASH) [44]. In the EAT, macrophage infiltration and adipocyte size were quantified using ImageJ software (imagej.net, NIH, Bethesda, MD, USA). The scoring criteria for each classification are shown in Table 1.

### 2.6. Quantification of Hepatic Lipid Levels

Hepatic lipids were extracted using a slightly modified version of the Bligh and Dyer method [45]. To quantify TG and TC in the liver, approximately 0.1 g of liver was homogenized in a chloroform (1:2, *v*/*v*) solution, followed by centrifugation at 805× *g* for 15 min. Next, the lower phase was carefully collected using a Pasteur pipette, and 1 mL *n*-hexane (3:2, *v*/*v*) was added to solubilize the lipids. The concentrations of TG and TC were measured using commercial kits (Embiel), and the results are expressed as mg per g of tissue weight.

### 2.7. Enzyme-Linked Immunosorbent Assay (ELISA)

An ELISA was used to quantify the serum levels of insulin (Mercodia, Uppsala, Sweden) and adipokine (leptin and adiponectin) in the EAT. EAT was treated using radioimmunoprecipitation assay (RIPA) buffer (ATTO, Tokyo, Japan) containing protease and phosphatase inhibitors (Thermo Fisher Scientific, Waltham, MA, USA). Thereafter, the lysates were analyzed using commercial ELISA kits (R&D Systems, Minneapolis, MN, USA). ELISA procedures were conducted according to the manufacturer’s instructions. The homeostatic model assessment of insulin resistance (HOMA-IR) was performed using the following established formula: (HOMA-IR = serum insulin (µg/L) × serum glucose (mg/dL)/405 [5].

### 2.8. Quantitative Reverse Transcription-Polymerase Chain Reaction (qRT-PCR)

For mRNA analysis of the liver and EAT, total RNA was extracted using the NucleoZoL reagent (Macherey-Nagel, GmbH & Co. KG, Duren, Germany) as described previously [46]. Subsequently, 2 µg mRNA was used to synthesize cDNA using an iScriptTM cDNA synthesis kit (Bio-Rad Laboratories, Hercules, CA, USA). Afterward, the cDNA was combined with iQTM SYBR Green Supermix (Bio-Rad Laboratories), and qRT-PCR was performed using a CFX96 Real-Time PCR Detection System (Bio-Rad Laboratories). The relative expression of each gene was determined using the 2ΔΔCT method, with mouse Gapdh serving as the reference gene. The primers used in the qRT-PCR targeted the expression of lipolytic and adipogenic enzymes. The primers used are listed in Table 2 and previous studies [47].

### 2.9. Western Blot Analysis

Western blot analysis was conducted based on previous research [48]. Briefly, liver tissue proteins were isolated using a Branson 450 Digital Sonifier Homogenizer (Branson, Danbury, CT, USA) in ice-cold RIPA lysis buffer (ATTO) containing protease and phosphatase inhibitors (Thermo Fisher Scientific). Next, equal amounts of protein (30 μg) were separated using 10% sodium dodecyl sulfate-polyacrylamide gel electrophoresis and transferred onto polyvinylidene difluoride membranes (Bio-Rad Laboratories). After blocking with 5% skim milk (Difco; BD Biosciences, Franklin Lakes, NJ, USA), the membranes were incubated with primary antibodies against phospho-AMP-activated protein kinase (*p*-AMPK [#2531]; Cell Signaling Technology, Danvers, MA, USA), total-AMP-activated protein kinase (t-AMPK [#2532]; Cell Signaling Technology), and glyceraldehyde-3-phosphate dehydrogenase (GAPDH [#sc-365062]; Santa Cruz, Dallas, TX, USA) overnight at 4 °C. Subsequently, the membranes were washed and exposed to secondary antibodies for 1 h at room temperature. After secondary antibody treatment, a chemiluminescent substrate solution (Thermo Fisher Scientific) was used for visualization using an Azure Biosystems Imaging System (Azure Biosystems, Dublin, CA, USA). Blot intensity was quantitatively analyzed using ImageJ Software (v.1.8; National Institutes of Health, Bethesda, MD, USA) (Table 3).

### 2.10. Statistical Analysis

The results are reported as means with their corresponding standard deviations (SDs). All figures were generated using GraphPad Prism 5 (GraphPad Software, Inc., San Diego, CA, USA). Statistical analysis was conducted using a one-way ANOVA followed by Tukey’s post hoc test, for comparing the NCD and HFD groups. Additionally, comparisons were made among the HFD, LL, and PC groups. The analysis was performed using XLAST 2012 statistical software (Addinsoft Inc., Paris, France). Statistical significance was set at *p* < 0.05 [49].

## 3. Results

### 3.1. Effects of LL on Body Weight (BW) and Food Efficiency

**Figure 2:**
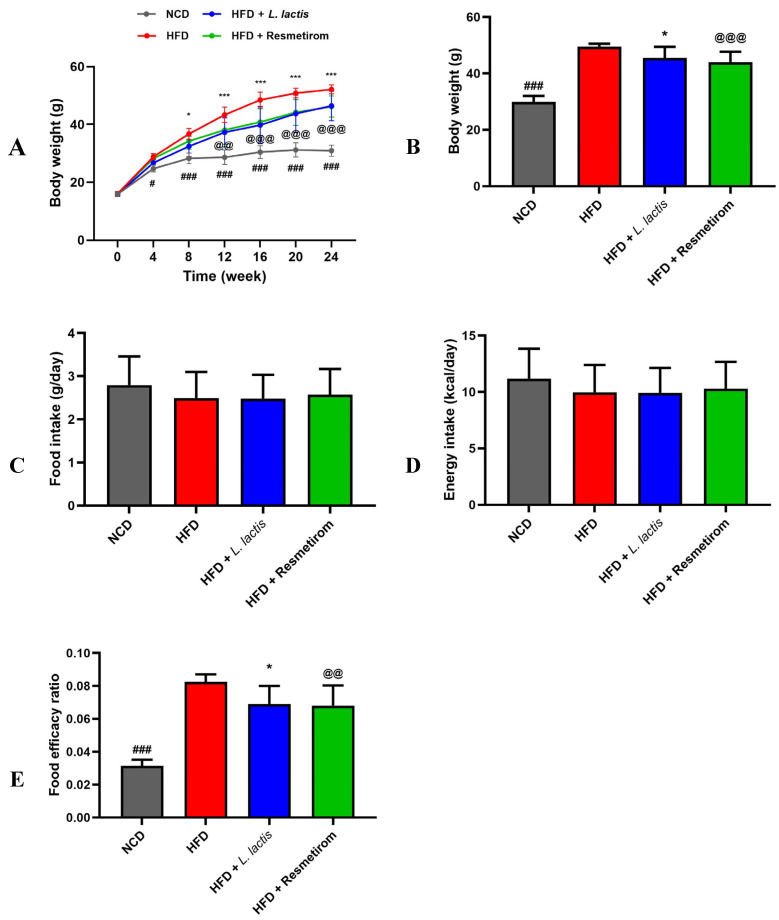
Effects of *L. delbrueckii* subsp. *lactis* CKDB001 on the body weight, final body weight, daily feed intake, energy intake, and food efficiency ratio of mice. The mice were subjected to the following dietary interventions for 24 weeks: normal chow diet (NCD), high-fat diet (HFD), HFD with *L. delbrueckii* subsp. *lactis* CKDB001 (LL), and HFD with resmetirom (positive control). (**A**) Weekly body weight over 24 weeks induced by HFD; (**B**) final body weight; (**C**) daily food intake; (**D**) energy intake; (**E**) food efficiency ratio. Values are presented as mean ± standard deviation (SD), and organ weights are expressed as a percentage of body weight (% of BW). # *p* < 0.05, ### *p*< 0.0001 between NCD and HFD; * *p* < 0.05, *** *p* < 0.001 between HFD and LL; @@ *p* < 0.01, @@@ *p* < 0.001 between HFD and PC.

Experimental C57BL/6J mice were fed either an NCD or HFD for a total of 24 weeks. After 12 weeks of designated dietary feeding, the mice were orally administered PBS, *L. lactis* CKDB001, or resmetirom to evaluate the effects of *L. lactis* CKDB001 in response to HFD consumption. Changes in BW over the 24-week dietary intervention period are shown in Figure 2A. In the HFD, BW noticeably increased by 4 weeks after dietary intervention. In contrast, BW decreased significantly in the LL group compared with that in the HFD. PC showed a significant reduction in BW compared with that in the HFD group from week 12 onward. Consequently, final BW was higher in the HFD than in the NCD group, whereas it was 0.92- and 0.89-fold lower in the LL and PC groups, respectively, although these differences were not statistically significant (Figure 2B). However, there were no significant differences in daily food intake and energy intake between the groups. According to the food efficiency ratio (FER) assessment, the NCD group had the lowest FER, whereas the HFD group had the highest FER. However, the LL and PC groups had remarkably lower FERs compared with that of the HFD group (Figure 2E).

### 3.2. Effect of LL on Liver and WAT Weights

**Figure 3:**
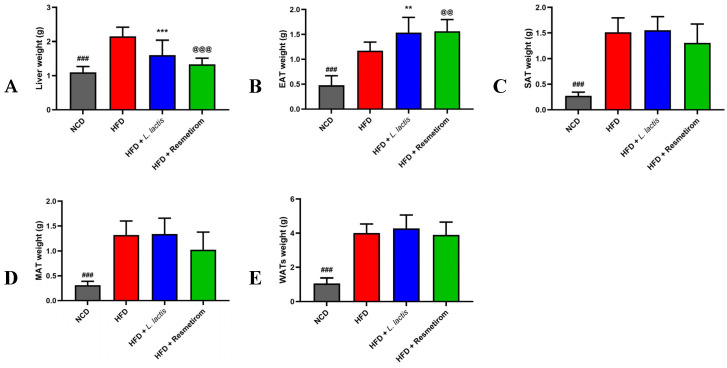
Effects of *L. delbrueckii* subsp. *lactis* CKDB001 on mouse organ weights. The mice were subjected to the following dietary interventions for 24 weeks: normal chow diet (NCD), high-fat diet (HFD), HFD with *L. delbrueckii* subsp. *lactis* CKDB001 (LL), and HFD with resmetirom (positive control). (**A**) Liver, (**B**) EAT, (**C**) SAT, (**D**) MAT, and (**E**) WATs weights. Values are presented as mean ± standard deviation (SD). ### *p* < 0.001 between NCD and HFD; ** *p* < 0.01, *** *p* < 0.001 between HFD and LL; @@ *p* < 0.01, @@@ *p* < 0.001 between HFD and PC. Abbreviation: EAT, epididymal adipose tissue; SAT, subcutaneous adipose tissue; MAT, mesenteric adipose tissue; WAT, white adipose tissue.

In metabolic complications, increased liver weight and excessive adipose tissue accumulation are associated with several health risks, including inflammation, insulin resistance, and dyslipidemia [50]. To assess changes in the weight of central metabolic organs, the weights of the liver and WATs (EAT, SAT, and MAT) were measured and presented in Figure 3 as a percentage of BW. In the HFD group, liver weight was significantly higher than that in the NCD group. However, LL and PC treatment resulted in significantly lower liver weights [0.75- (*p* < 0.01) and 0.63-fold (*p* < 0.001), respectively, compared with that in the HFD group (Figure 3A). In the HFD group, the relative weight of EAT was higher than that of the NCD group. Notably, EAT weight in the LL and PC groups was 1.29- and 1.22-fold higher, respectively, than that in the HFD group (*p* < 0.05; Figure 3B). Furthermore, the weights of SAT, MAT, and total WATs in the HFD group were significantly higher than those in the NCD group (*p* < 0.0001), but they did not significantly differ between the HFD and LL or PC groups (Figure 3C–E). These results suggest that LL consumption could prevent HFD-induced hepatomegaly, but do not markedly affect overall fat weight gain.

### 3.3. LL Maintains Intact Insulin Sensitivity in HFD-Induced Mice

**Figure 4:**
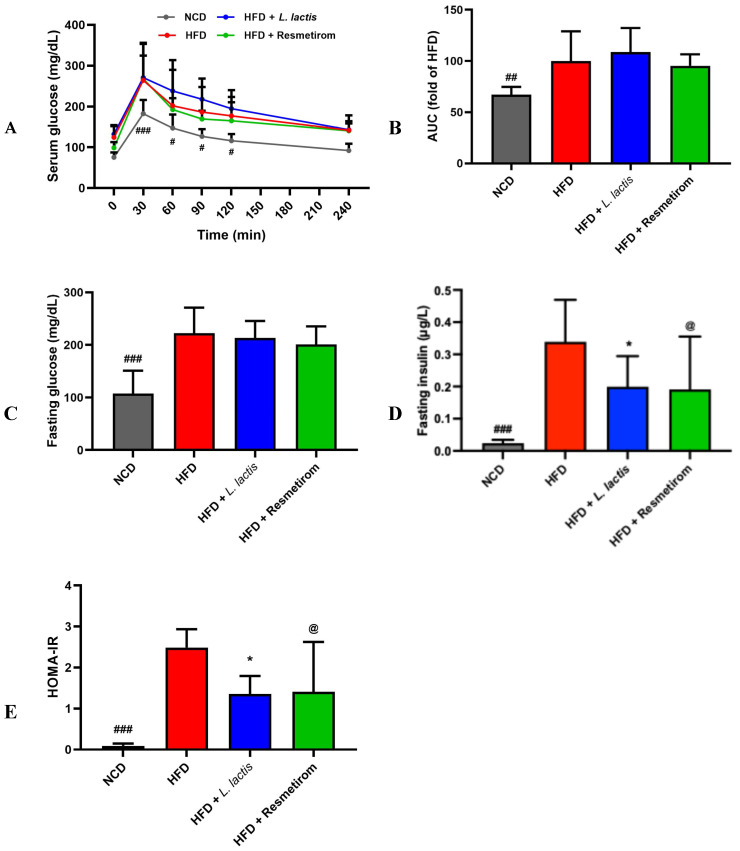
Effects of *L. delbrueckii* subsp. *lactis* CKDB001 on serum glucose levels of mice. The mice were subjected to the following dietary interventions for 24 weeks: normal chow diet (NCD), high-fat diet (HFD), HFD with *L. delbrueckii* subsp. *lactis* CKDB001 (LL), and HFD with resmetirom (positive control). (**A**) Serum glucose; (**B**) AUC of OGTT; (**C**) fasting glucose; (**D**) fasting insulin; (**E**) HOMA-IR. Values are presented as mean ± standard deviation (SD). # *p* < 0.05, ## *p* < 0.01, ### *p* < 0.001 between NCD and HFD; * *p* < 0.05 between HFD and LL; @ *p* < 0.05 between HFD and PC. Abbreviation: AUC, area under the curve; OGTT, oral glucose tolerance test; HOMA-IR, homeostasis model assessment-estimated insulin resistance.

To assess the impact of LL supplementation on insulin resistance, we conducted OGTTs. The results showed significantly higher glucose levels in the HFD group compared with those in the NCD group, but those in the HFD, LL, and PC groups did not significantly differ (Figure 4A,B). Similarly, the serum glucose levels in the HFD group were significantly higher than those in the NCD group (*p* < 0.05). The serum glucose levels in the LL and PC groups were 0.94- and 0.88-fold lower, respectively, relative to those in the HFD group, but these differences were not significant (Figure 4C). Additionally, the HFD group displayed 10.96-fold higher serum insulin levels than those in the NCD group (*p* < 0.001). However, the insulin levels in the LL and PC groups were 0.64- and 0.60-fold lower than those in the HFD group, respectively (*p* < 0.05; Figure 4D). Consequently, the HOMA-IR calculated from glucose and insulin levels was significantly lower in the NCD group compared with that in the HFD group. Similarly, both LL and PC groups also showed significantly lower levels (*p* < 0.05; Figure 4E). Accordingly, while LL consumption may show potential for improving insulin sensitivity against HFD-induced metabolic syndrome by regulating pancreatic insulin secretion, the current results did not reveal statistically significant differences in serum glucose levels.

### 3.4. LL Attenuates CVD Risk Markers in HFD-Induced Mice

**Figure 5:**
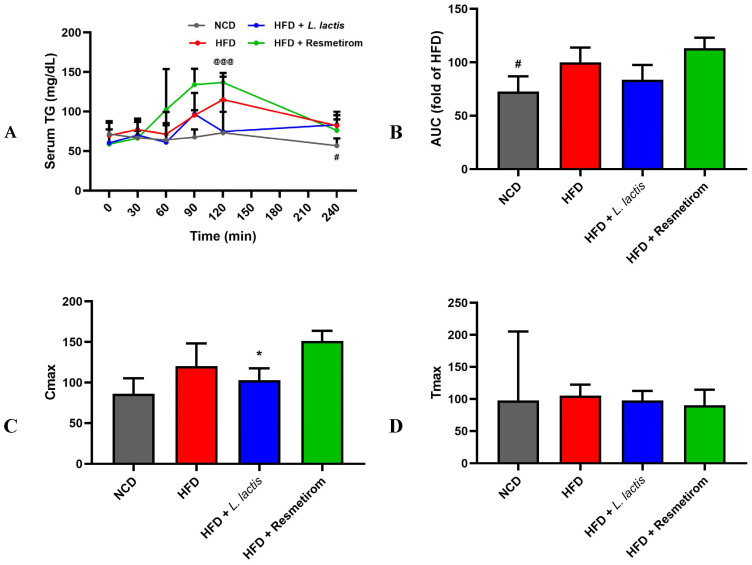

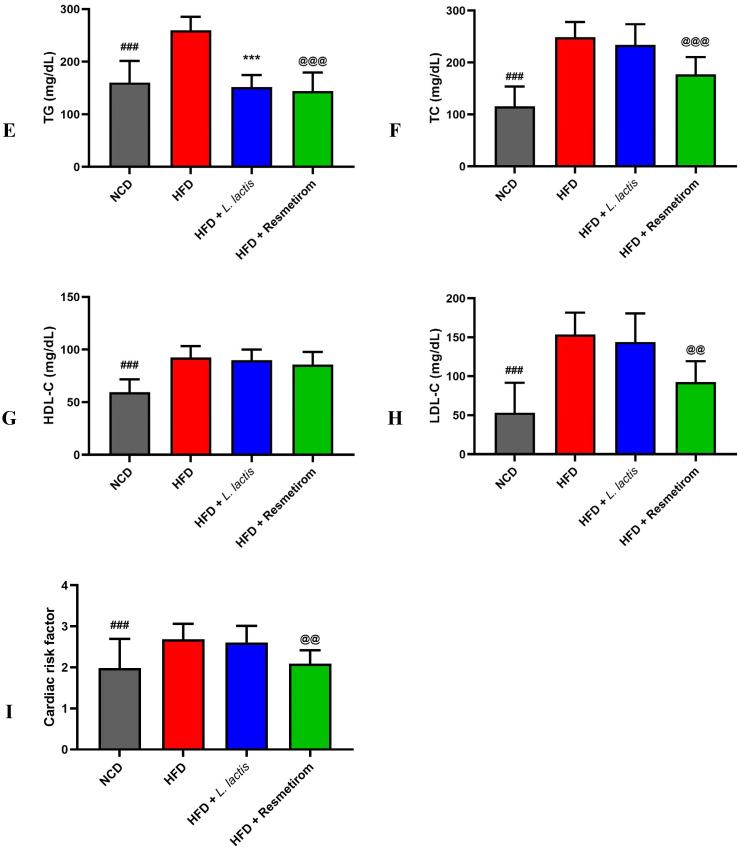
Effects of *L. delbrueckii* subsp. *lactis* CKDB001 on serum TC, TG, HDL-c, LDL-c, and CRF in mice. The mice were subjected to the following dietary interventions for 24 weeks: normal chow diet (NCD), high-fat diet (HFD), HFD with *L. delbrueckii* subsp. *lactis* CKDB001 (LL), and HFD with resmetirom (positive control). (**A**) Serum TG; (**B**) AUC of OLTT; (**C**) Cmax; (**D**) Tmax; (**E**) TG; (**F**) serum TC; (**G**) serum HDL-cholesterol; (**H**) serum LDL-cholesterol; (**I**) cardiac risk factor. Values are presented as mean ± standard deviation (SD). # *p* < 0.05, ### *p* < 0.001 between NCD and HFD; * *p* < 0.05, *** *p* < 0.001 between HFD and LL; @@ *p* < 0.01, @@@ *p* < 0.001 between HFD and PC. Abbreviation: TG, triglyceride; AUC, area under the curve; OLTT, oral lipid tolerance test; TC, total cholesterol; HDL, high-density lipoprotein; LDL, low-density lipoprotein.

To determine whether LL prevents hyperlipidemia induced by an HFD, we conducted OLTTs. The results showed that blood lipid levels were significantly higher in the HFD group compared with those in the NCD group. Although the LL group showed 0.90-fold lower blood lipid levels than those in the HFD group, the difference did not reach statistical significance (Figure 5A,B). Additionally, Cmax was 0.80- and 0.95-fold lower in the NCD and LL groups, respectively, than in the HFD group, but these differences were also not significant. However, the PC group exhibited a 1.39-fold higher Cmax than that of the HFD group (Figure 5C). The Tmax was similar across all groups, with no notable differences (Figure 5D).

Furthermore, to evaluate the effects of LL on lipid reduction and CVD risk factors in diet-induced dyslipidemia, we conducted lipid panel assessments and analyzed CRFs through biochemical serum analysis. Serum TG and TC levels in the HFD group were 1.61- and 2.18-fold higher than those in the NCD group (Figure 5E,F). Notably, serum TG levels were 0.59- and 0.57-fold lower in the LL and PC groups, respectively, compared with those in the HFD group (*p* < 0.001; Figure 5E). However, while serum TC levels were 0.72-fold lower in the PC group (*p* < 0.001), LL did not reduce TC levels (Figure 5F).

Serum HDL-C levels were significantly higher in the HFD group compared with those in the NCD group (*p* < 0.001), but they did not significantly differ between the HFD, LL, and PC groups (Figure 5G). LDL-C levels were significantly higher in the HFD than in the NCD group (*p* < 0.001). LL did not significantly lower LDL-C levels relative to those in the HFD group, but those in the PC group were 0.60-fold lower (Figure 5H). Similarly to the LDL-C results, CRF levels were higher in the HFD than in the NCD group, significantly contributing to dyslipidemia (*p* < 0.05; Figure 5I). While LL tended to decrease CRF levels, significance was not reached; however, the PC group exhibited significantly lower levels.

In summary, our findings indicate that LL supplementation may improve catabolic lipid metabolism or enhance the clearance of TG from the bloodstream, potentially reducing the risk of associated metabolic disorders. However, LL did not significantly reduce TC or LDL-C levels. Therefore, while LL may have potential lipid-lowering effects and contribute to cardiovascular homeostasis, further research under different pathologic experimental conditions is necessary to confirm these findings.

### 3.5. LL Ameliorates Hepatic Lipid Accumulation and Function in HFD-Induced Mice

**Figure 6:**
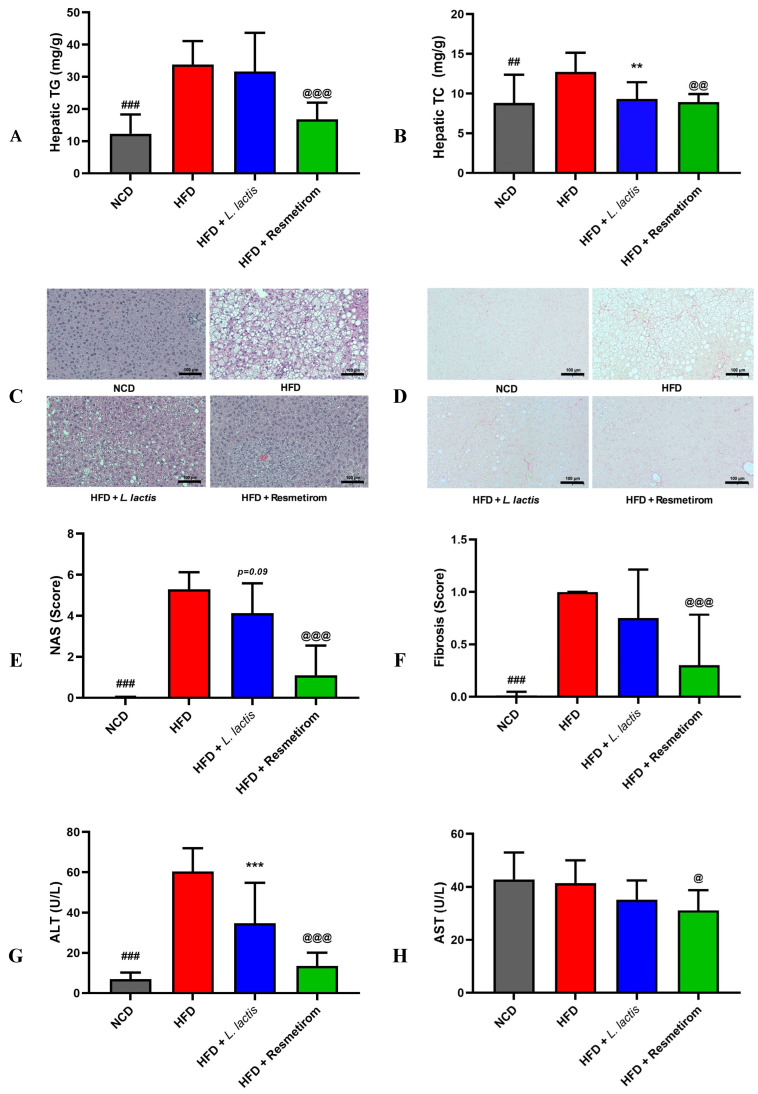
Effects of *L. delbrueckii* subsp. *lactis* CKDB001 on serum hepatic TG, hepatic TC, histology, NAS, fibrosis, ALT, and AST levels of mice. The mice were subjected to the following dietary interventions for 24 weeks: normal chow diet (NCD), high-fat diet (HFD), HFD with *L. delbrueckii* subsp. *lactis* CKDB001 (LL), and HFD with resmetirom (positive control). (**A**) Hepatic TG; (**B**) hepatic TC. (**C**) H&E and (**D**) sirius red staining images of the liver. (**E**) NAS; (**F**) hepatic fibrosis; (**G**) serum ALT; (**H**) serum AST. Values are presented as mean ± standard deviation (SD). ## *p* < 0.01, ### *p* < 0.001 between NCD and HFD; ** *p* < 0.01, *** *p* < 0.001 between HFD and LL; @ *p* < 0.05, @@ *p* < 0.01, @@@ *p* < 0.001 between HFD and PC. Abbreviations: TG; triglyceride, TC; total cholesterol, H&E; hematoxylin and eosin, NAS; NAFLD activity score, ALT; alanine aminotransferase, AST; aspartate aminotransferase.

To determine whether systemic metabolic disorders induced by HFD intake cause pathological stress in hepatocytes, we measured hepatic lipid levels, histological characteristics, and liver enzyme activities. Hepatic TG and TC levels were 2.61- (*p* < 0.001) and 1.41-fold (*p* < 0.05) higher in the HFD group, respectively, relative to those in the NCD group (Figure 6A,B). In the PC group, TG and TC levels were 0.49- and 0.72-fold lower, respectively (*p* < 0.001). In the LL group, TG and TC levels were 0.92- and 0.76-fold lower, respectively, but only the decrease in TG levels was statistically significant (Figure 6A,B).

Histological evaluation of the liver using H&E staining revealed severe vacuolation in the HFD group, which was characterized by micro- and macrovascular steatosis due to ectopic WAT deposition in hepatocytes. Contrastingly, visual inspection showed reduced steatosis and fibrosis in the LL and PC groups compared with those in the HFD group (Figure 6C,D). Quantification of NAS and fibrosis from hepatic histological analysis showed significantly higher levels in the HFD than in the NCD group. In the LL group, NAS and fibrosis showed a decreasing trend compared with that in the HFD group, but the differences were not significant (Figure 6E,F). However, the PC group showed significantly lower NAS and fibrosis than those in the HFD group (Figure 6E,F).

Furthermore, serum ALT and AST activities were examined to evaluate the effects of LL on liver enzymatic function. The ALT levels were significantly higher in the HFD than in the NCD group (*p* < 0.001; Figure 6G). However, the LL and PC groups exhibited significantly lower ALT levels (0.57- and 0.22-fold, respectively) than those in the HFD group (Figure 6G). AST levels did not significantly differ between the dietary intervention groups, except for the PC group (Figure 6H). Therefore, systemic metabolic disorders induced by HFD intake resulted in hepatic lipid accumulation, increased liver injury markers, and worsened histological characteristics. Hepatic pathology caused by HFD consumption was mitigated by LL and PC interventions, as evidenced by reductions in hepatic TG and TC levels, improved histological features, and decreased ALT levels.

### 3.6. LL Modulates AMPK and Lipid Metabolism-Related Gene Expression in the Liver

**Figure 7:**
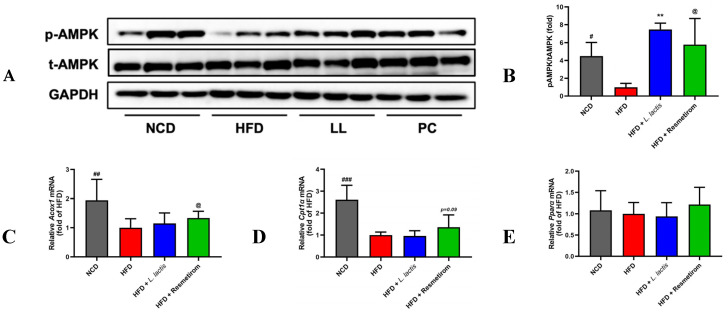

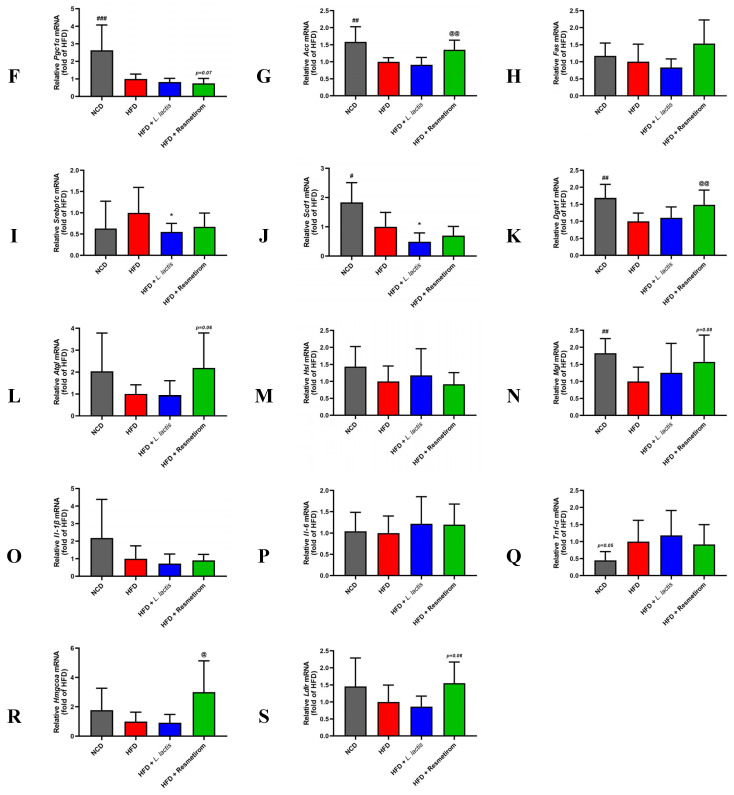
Effects of *L. delbrueckii* subsp. *lactis* CKDB001 on hepatic AMPK activity and mRNA levels associated with mouse lipid metabolism. The mice were subjected to the following dietary interventions for 24 weeks: normal chow diet (NCD), high-fat diet (HFD), HFD with *L. delbrueckii* subsp. *lactis* CKDB001 (LL), and HFD with resmetirom (positive control). (**A**) Representative Western blot images. (**B**) *p*-AMPK levels; relative gene expression of (**C**) *Acox1*; (**D**) *Cpt1α*; (**E**) *Pparα*; (**F**) *Pgc1α*; (**G**) *Acc*; (**H**) *Fas*; (**I**) *Srebp1c*; (**J**) *Scd1*; (**K**) *Dgat1*; (**L**) *Atgl*; (**M**) *Hsl*; (**N**) *Mgl*; (**O**) *Il-1β*; (**P**) *Il-6*; (**Q**) *Tnf-α*; (**R**) *Hmg-coa reductase*; (**S**) *Ldlr*. Values are presented as mean ± standard deviation (SD). # *p* < 0.05, ## *p* < 0.01, ### *p* < 0.001 between NCD and HFD; * *p* < 0.05, ** *p* < 0.01 between HFD and LL; @ *p* < 0.05, @@ *p* < 0.01 between HFD and PC. Abbreviation: *p*-AMPK, phospho-AMP-activated protein kinase; *Acox1*, Peroxisomal acyl-coenzyme A oxidase 1; *Cpt1α*, Carnitine palmitoyl transferase I; *Pparα*, Peroxisome proliferator-activated receptor alpha; *Pgc1α*, Peroxisome proliferator-activated receptor gamma coactivator 1 alpha; *Acc*, Acetyl-CoA carboxylase; *Fas*, fatty acid synthase; *Srebp1c*, Sterol regulatory element-binding protein-1c; *Scd1*, Stearoyl-CoA 9-desaturase; *Dgat1*, Diacylglycerol O-acyltransferase 1; *Atgl*, Adipose triglyceride lipase; *Hsl*, hormone-sensitive lipase; *Mgl*, Monoacylglycerol lipase; *Il*, Interleukin; *Tnf-α*, tumor necrosis factor alpha; *Hmg-coa*, Hydroxymethylglutaryl-CoA reductase; *Ldlr*, low-density lipoprotein receptor.

The HFD-induced metabolic disturbances in energy and lipid metabolism in the liver may lead to various pathological progressions [51]. However, LL treatment significantly mitigated HFD-induced metabolic disturbances; therefore, we logically postulated that LL treatment would also alter energy and lipid metabolism beneficially. Therefore, the expression levels of energy and lipid metabolism-related biomarkers in the liver were measured following HFD intake. The *p*-AMPK levels in the liver were 4.50-fold lower in the HFD than in the NCD group (*p* < 0.05; Figure 7A,B). Notably, LL and PC treatments resulted in 7.47- (*p* < 0.001) and 5.79-fold (*p* < 0.05) higher *p*-AMPK levels, respectively, compared with those in the HFD group (Figure 7A,B). Additionally, the expression levels of genes involved in fatty acid β-oxidation were measured to determine the effects of LL on lipid metabolism. Anabolic gene expression related to de novo ectopic adipogenesis, including *Acox1*, *Cpt1α*, *Pparα*, *Pgc1α*, *Acc*, *Fas*, *Srebp1c*, *Scd1*, and *Dgat1*, was measured by qRT-PCR. The HFD groups exhibited significantly lower expressions of adipogenic genes, such as *Acox1*, *Cpt1α*, *Pgc1α*, *Acc*, *Scd1*, and *Dgat1*, reaching levels as low as 51.5%, 38.3%, 38.0%, 63.0%, 54.5%, and 59.3%, respectively, compared to those in the NCD group (Figure 7C–K). Interestingly, among the anabolic gene expressions, hepatic *Scd1* expression in the LL group was significantly decreased compared to the HFD group (Figure 7J). Furthermore, hepatic *Srebp1c* expression in the LL group (~54%) was significantly lower than in the HFD group, which was elevated by HFD consumption compared to the NCD, *Il-6*, *Tnf- α*, *Hmgcoa reductase*, and *Ldlr* in the liver were analyzed by qRT-PCR (Figure 7J,P,Q,S). However, LL did not significantly alter lipolysis, cytokine, or cholesterol regulatory gene expressions. These results suggest that LL treatment significantly improves *p*-AMPK levels and influences gene expression associated not with catabolic, but with anabolic responses in lipid metabolism, including lipid synthesis. Our findings imply potential therapeutic benefits in managing HFD-induced metabolic disturbances in the liver.

### 3.7. LL Inhibits Lipid Accumulation and Modulates Leptin and Adiponectin Levels in HFD-Induced Mice

**Figure 8:**
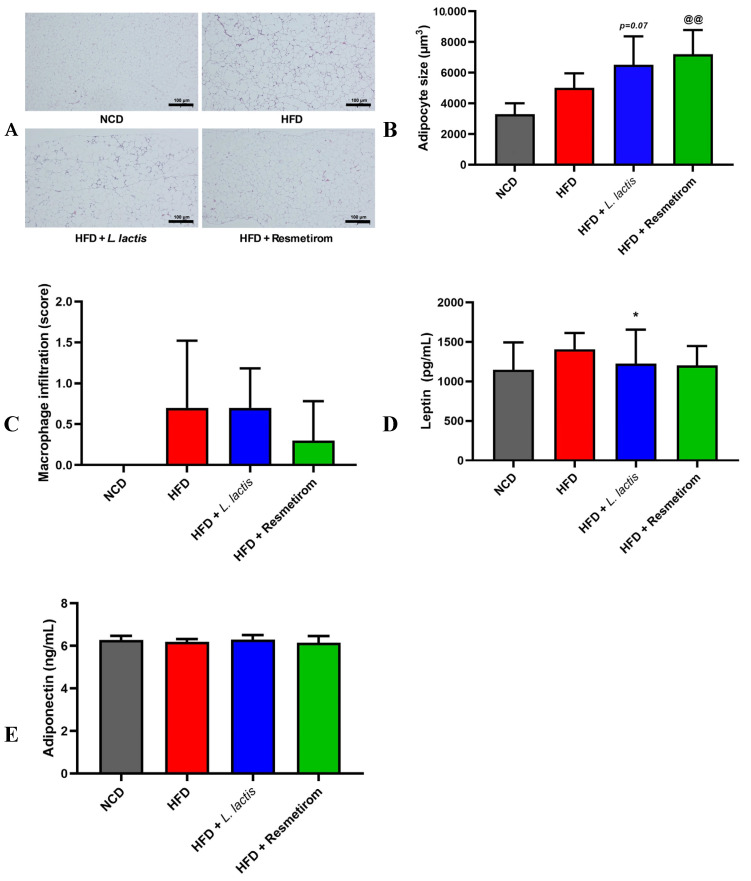
Effects of *L. delbrueckii* subsp. *lactis* CKDB001 on mouse lipid metabolism. The mice were subjected to the following dietary interventions for 24 weeks: normal chow diet (NCD), high-fat diet (HFD), HFD with *L. delbrueckii* subsp. *lactis* CKDB001 (LL), and HFD with resmetirom (positive control). (**A**) H&E staining image of EAT. (**B**) Adipocyte size in EAT. (**C**) Macrophage infiltration; (**D**) Leptin; (**E**) Adiponectin. Values are presented as mean ± standard deviation (SD). * *p* < 0.05 between HFD and LL; @@ *p* < 0.01 between HFD and PC. Abbreviation: H&E staining image of EAT; EAT, epididymal adipose tissue.

Epididymal adipose tissue (EAT) is a crucial endocrine organ that regulates energy metabolism and immune responses. Excessive EAT accumulation due to an HFD is closely linked to various metabolic complications [52]. Histological analysis of EAT revealed that adipocyte size in the HFD group was significantly higher (1.69-fold) than that in the NCD group, while LL and PC treatment led to significantly higher adipocyte sizes (1.25- and 1.23-fold, respectively) compared with those in the HFD group (Figure 8A,B). However, macrophage infiltration among the groups did not significantly differ (Figure 8C). Additionally, the effect of LL on the expression of leptin and adiponectin, hormones involved in energy balance and metabolic regulation, was examined in the lysates of EAT. Although no significant differences were observed in leptin and adiponectin levels between the NCD and HFD groups, leptin expression was 0.84-fold lower in the LL than in the HFD group (*p* < 0.05, Figure 8D,E). Collectively, these findings indicate that LL treatment further induced the increased adipocyte size in EAT caused by an HFD. Despite no significant differences in macrophage infiltration or adiponectin levels, LL treatment notably reduced leptin expression, suggesting potential benefits in managing HFD-induced metabolic disturbances in EAT.

## 4. Discussion

Probiotics have emerged as a promising therapeutic approach for patients with MASLD, formerly known as NAFLD. Probiotics play a crucial role in restoring gut microbiota balance by increasing the abundance of beneficial bacteria and reducing that of harmful bacteria in the gut [53]. The gut microbiota, consisting of trillions of microorganisms in the gastrointestinal tract, are essential for maintaining host health and homeostasis, with compositional changes playing a major role in various diseases [54,55]. Recent studies have highlighted the involvement of the gut microbiota in liver homeostasis, particularly via regulation of the gut–liver axis [56]. The liver and gut communicate primarily via two pathways—the hepatic portal vein and bile acids (BAs) [57]. The hepatic portal vein carries most of the blood from the intestines to the liver, while BAs transport metabolites from the liver to the intestines [58]. Owing to the intertwined physiological relationship between the gut and liver, alterations in the gut microbiome by probiotics have gained attention as a potential therapeutic approach for liver diseases, including MASLD [59].

Additionally, probiotics have demonstrated positive effects on metabolic parameters associated with MASLD, such as improved insulin sensitivity [60,61] and lipid metabolism [62,63]. These metabolic improvements are essential for preventing or managing MASLD, as the condition is closely linked to metabolic dysfunction. Some studies have suggested that probiotics may serve as an alternative to antibiotics in MASLD treatment [64]. For instance, certain probiotic strains, such as *Lactococcus lactis*, have shown promise in mitigating MASLD by reducing lipid accumulation and improving liver health [65,66]. Previous research demonstrated that *L. delbrueckii* subsp. *lactis* CKDB001 reduces lipid accumulation and improves the lipid profiles of hepatic cells [35]. Moreover, Lee et al. found that *L. delbrueckii* subsp. *lactis* CKDB001 alleviated the Western diet-induced NAFLD by improving the liver-to-BW ratio, reducing steatosis, and lowering inflammation grade and NAS in the liver [36]. However, despite these promising results, the precise mechanisms through which *L. delbrueckii* subsp. *lactis* CKDB001 affects lipid metabolism remain unclear. Furthermore, issues such as low bioavailability and limited clinical data still hinder the use of *L. delbrueckii* subsp. *lactis* CKDB001 in the treatment of patients with MASLD [67,68].

A high dietary fat intake facilitates excessive caloric accumulation, elevating the risk of obesity by promoting WAT deposition [69]. Diets high in saturated fat, such as HFDs, are major contributors to obesity due to their high energy density. Obesity, in turn, is associated with an increased risk of metabolic disorders, including dyslipidemia, insulin resistance, and CVD [70]. Several studies have established obesity models in experimental mice using HFDs, which induce ectopic fat accumulation in the liver and BW gain [71]. Furthermore, HFD consumption ultimately leads to metabolic diseases such as hepatic steatosis, hypertension, and insulin resistance [72]. Previous research has demonstrated that HFDs, particularly those comprising 60% fat, are linked to obesity, hepatomegaly, and compromised liver function [73]. The existing studies primarily focused on reducing calorically dense fat intake to manage obesity while exploring nutraceuticals that may prevent obesity and its metabolic complications. Contrastingly, the present study aimed to investigate the impact of *L. delbrueckii* subsp. *lactis* CKDB001 on insulin resistance, dyslipidemia, MASLD, and lipid metabolism in an HFD-induced obese mouse model.

We investigated the effects of *L. delbrueckii* subsp. *lactis* CKDB001 on obesity and assessed its protective effects against the progression of metabolic disorders associated with obesity. BW is commonly used as a primary indicator of obesity [74], and our findings demonstrated that HFD-fed mice showed significant weight gain compared with those on an NCD. However, LL supplementation resulted in a notable reduction in BW. Additionally, LL supplementation reduced food efficiency, meaning that despite increased food intake, weight gain was less, indicating effective weight management. HFD-induced obesity and overweight are closely linked to peripheral insulin resistance and hyperinsulinemia, both of which contribute to metabolic disorders [75]. In the present study, HFD intake markedly elevated fasting blood glucose levels along with serum insulin levels compared with those in the NCD group. Notably, LL administration reduced the serum insulin levels in HFD-fed mice. These findings suggest that LL reduced HOMA-IR levels, a widely used indicator for assessing β-cell function and insulin resistance under steady-state conditions [76]. Elevated HOMA-IR values are associated with reduced insulin sensitivity, indicating insulin resistance [77]. The observed reduction in HOMA-IR levels suggests that LL treatment may effectively prevent or mitigate insulin resistance induced by HFD. However, no significant changes in blood glucose levels were observed with LL treatment, possibly due to the short dietary intervention period of this study. Previous studies have shown reduced blood glucose levels with longer HFD feeding periods, such as over 30 weeks [78]. Therefore, future studies should consider longer intervention durations to fully understand the effects of LL on glucose homeostasis.

Insulin resistance plays a key role in the development of dyslipidemia, even in individuals with normal glucose tolerance [79]. Dyslipidemia associated with insulin resistance is typically characterized by elevated TG and LDL-C levels, along with reduced HDL-C levels, commonly referred to as the “atherogenic lipid triad”, which considerably increases the risk of CVD [79,80]. To evaluate the effects of LL on dyslipidemia, serum TG and TC levels were analyzed. HFD feeding led to higher TG and TC levels than those in the NCD group, but LL treatment reduced TG levels without significantly affecting TC levels. Consistent with these findings, other studies have also reported that *L. lactis* treatment reduces serum TG levels [81]. The reduction in TG levels is possibly due to the ability of LL to modulate the gut microbiota, which inhibits the growth of harmful bacteria while promoting beneficial bacteria, thereby improving gut health and reducing serum TG levels. Nevertheless, future studies are necessary to examine changes in the distribution of gut microbiota to better understand the interaction between LL, gut microbiota, and lipid metabolism. Although LL supplementation tended to reduce TC, LDL-C, and CRF levels, this reduction was not statistically significant relative to that in the HFD group. HFD feeding also led to increased HDL-C levels, which may be linked to elevated free cholesterol (FC) levels [82]. Rosales et al. recently suggested that high plasma concentrations of HDL-C, combined with elevated FC content, may facilitate the net transfer of FC into tissues, potentially leading to an atherogenic process [78]. Since FC movement between lipid surfaces is reversible, increased HDL-FC bioavailability could result in the deposition of excess FC in cells in vivo [83]. Future studies should focus on how FC levels are modulated by LL treatment.

MASLD is diagnosed when more than 5% of the liver has ectopic fat [84]. The pathogenesis of MASLD is often explained by the “two-hit hypothesis” [85]. The first hit involves the accumulation of lipids in hepatocytes, primarily TG, leading to steatosis. The second hit encompasses various factors that promote injury, inflammation, and fibrosis [86]. Persistent ectopic fat accumulation in the liver may lead to hepatic inflammation, contributing to metabolic dysfunction-associated steatohepatitis (MASH) development [57]. The progression from simple steatosis to MASH aligns with the second hit in the hypothesis. Prolonged MASLD or MASH, accompanied by inflammatory responses, may progress to liver fibrosis, cirrhosis, or hepatocellular carcinoma [87]. Preventing ectopic fat accumulation in the liver is crucial for effectively managing and treating MASLD, as it addresses the initial hit in disease progression [88]. In the present study, HFD consumption led to increased liver weight and hepatic TG and TC levels, indicating ectopic fat accumulation and the successful establishment of a MASLD model. LL consumption resulted in reduced liver weight and hepatic TC levels, suggesting a liver-specific effect on cholesterol metabolism. The lipid-lowering effect of LL treatment may be due to enhanced excretion of cholesterol, inhibition of hepatic cholesterol synthesis, or improved clearance of cholesterol from the bloodstream. These effects could help mitigate fatty liver disease and protect against liver cell lipotoxicity, potentially targeting both the first and second hits in MASLD pathogenesis. LL supplementation also attenuated NASs, hepatic fibrosis, and liver function indices such as AST. The decrease in hepatic TC levels may correlate with the reduction in NASs and fibrosis [89].

Moreover, HFD intake reduced AMPK phosphorylation in the liver, whereas LL treatment enhanced AMPK phosphorylation. AMPK plays a crucial role in regulating energy metabolism, acting as a cellular energy sensor and master regulator of metabolic homeostasis. AMPK activation promotes energy-producing pathways, such as fatty acid oxidation and glucose uptake, while inhibiting energy-consuming processes, such as fatty acid and protein synthesis [90,91,92]. The beneficial effects of AMPK activation, including improved insulin sensitivity [93,94,95], enhanced mitochondrial function [96], and reduced lipid accumulation [97,98,99] in the liver, contribute to improved liver function and reduced risk of MASLD. Experimental studies have demonstrated that both genetic and pharmacological activation of AMPK ameliorates fatty liver disease [100,101]. Accordingly, enhancing AMPK activity is considered a promising therapeutic approach for treating MASLD.

In the current study, LL intake protected against the HFD-induced reduction in hepatic AMPK phosphorylation, resulting in associated benefits in hepatic lipid metabolism. LL may have acted as an AMPK agonist, contributing to the reduction in ectopic fat accumulation in the liver. The observed reduction in liver weight and hepatic TC levels following LL consumption suggests that LL enhanced AMPK activity, potentially leading to increased fatty acid oxidation and decreased lipogenesis. The AMPK-induced effect played a crucial role in alleviating ectopic fat accumulation in the liver, thus counteracting MASLD development [102,103]. Notably, LL supplementation enhanced AMPK phosphorylation along with hepatic gene expressions related to de novo adipogenesis, such as Scd1 and Srebp1c. These findings suggest that the beneficial effects of LL on liver metabolism are primarily mediated through post-translational modifications and the suppressive transcriptional regulation of adipogenic gene expressions. Therefore, the beneficial effects of LL on liver metabolism may occur mainly via AMPK activation and subsequent post-translational modifications and changes in transcriptional expression.

MASLD primarily involves ectopic fat accumulation in the liver, but fat deposition in other tissues, such as EAT, also plays a crucial role in the progression of the disease [104]. EAT is metabolically sensitive and secretes adipokines such as leptin and adiponectin, which regulate metabolic homeostasis [105]. Under conditions of obesity and MASLD, adipokine secretion becomes imbalanced, promoting systemic inflammation and insulin resistance [106]. In the current study, LL treatment reduced leptin levels in EAT without significantly affecting adiponectin levels, consistent with previous findings [107]. The reduction in leptin levels is particularly relevant, as elevated leptin levels are linked to leptin resistance, a condition commonly observed in obesity and MASLD, which further exacerbates systemic inflammation and insulin resistance. A reduction in leptin levels may indicate improved leptin sensitivity, which could reduce inflammation and insulin resistance [108], thereby restoring metabolic balance and supporting beneficial outcomes in insulin sensitivity and lipid metabolism [109]. These findings are consistent with the broad effects of LL observed in the current study, including reduced BW, improved insulin sensitivity, and decreased serum TG levels.

Interestingly, histological analysis revealed that HFD intake increased adipocyte size, and notably, LL treatment also increased adipocyte size, potentially due to enhanced glucose uptake and mitochondrial function. The increase in adipocyte size following LL treatment suggests that LL supplementation induced hypertrophy in a metabolically healthier manner by promoting mitochondrial biogenesis and energy utilization, rather than simply increasing fat storage.

This study demonstrates the therapeutic potential of *L. delbrueckii* subsp. *lactis* CKDB001 (LL) in addressing metabolic dysfunctions associated with MASLD, including reductions in BW, improvements in insulin sensitivity, decreases in serum TG levels, and enhancements in AMPK phosphorylation in the liver. The comprehensive experimental design, utilizing a well-established HFD-induced murine model and detailed physiological and biochemical analyses, provides valuable insights into the role of *L. delbrueckii* subsp. *lactis* CKDB001 in lipid metabolism and gut microbiota modulation. However, the precise mechanisms underlying these effects remain unclear, and the short intervention duration may have limited observations of long-term outcomes. Challenges in translating findings to clinical applications are noted due to low bioavailability and limited human data. Additionally, while liver-specific effects were emphasized, further investigation into extrahepatic outcomes and direct evidence of gut microbiota interactions is needed to fully understand the therapeutic potential of *L. delbrueckii* subsp. *lactis* CKDB001.

## 5. Conclusions

In conclusion, LL supplementation ameliorated the HFD-induced metabolic dysregulation associated with MASLD, i.e., reduced hepatomegaly, decreased NASs and fibrosis, and enhanced AMPK phosphorylation in the liver. LL supplementation also modulated leptin levels, suggesting its potential role in regulating energy metabolism. These findings collectively highlight the potential of LL as a therapeutic candidate for ameliorating HFD-induced MASLD. However, several important areas require further investigation to fully understand the efficacy of LL as a potential probiotic supplement. Future studies should focus on assessing the bioavailability of LL in clinical settings and explore methods to enhance stability or absorption in the body. Additionally, as the present study lacks a detailed understanding of how LL affects specific bacterial strains and gut composition during gut microbiota modulation, a comprehensive investigation of gut microbiota changes is required.

## Figures and Tables

**Table 1:** Scoring system for histopathological assessment.

Item	Score	Extent
NAS	0	<5%
	1	5–33%
	2	34–66%
	3	>66%
Fibrosis	0	None
	1	Perisinusoidal or periportal
	1a	Mild, zone 3, perisinusoidal
	1b	Moderate, zone 3, perisinusoidal
	1c	Portal/periportal
	2	Perisinusoidal and portal/periportal
	3	Bridging fibrosis
	4	Cirrhosis
Macrophage infiltration	0	None
	1	Minimal (<10%)
	2	Mild (10–25%)
	3	Moderate (26–50%)
	4	Marked (>51%)

Abbreviation: NAS, non-alcoholic fatty liver disease activity score.

**Table 2:** qRT-PCR primer sequences (5′ to 3′).

Transcript	Forward	Reverse
*Acox1*	CAGGAAGAGCAAGGAAGTGG	CCTTTCTGGCTGATCCCATA
*Cpt1α*	CTCCGCCTGAGCCATGAAG	CACCAGTGATGATGCCATTCT
*Pparα*	GTACGGTGTGTATGAAGCCATCTT	GCCGTACGCGATCAGCAT
*Atgl*	TGTGGCCTCATTCCTCCTAC	TCGTGGATGTTGGTGGAGCT
*Hsl*	GCTGGGCTGTCAAGCACTGT	GTAACTGGGTAGGCTGCCAT
*Mgl*	CGGACTTCCAAGTTTTTGTCAGA	GCAGCCACTAGGATGGAGATG
*Acc*	TGGACAGACTGATCGCAGAGAAAG	TGGAGAGCCCCACACACA
*Fas*	GGAGGTGGTGATAGCCGGTAT	TGGGTAATCCATAGAGCCCAG
*Scd1*	TTCTTGCGATACACTCTGGTGC	CGGGATTGAATGTTCTTGTCGT
*Dgat1*	GAGTCTATCACTCCAGTGGG	GGCGGCACCACAGGTTGACA
*Pgc1α*	CCCAGGCAGTAGATCCTCTTCAA	CCTTTCGTGCTCATAGGCTTCATA
*Srebp1*	GCAGCCACCATCTAGCCTG	CAGCAGTGAGTCTGCCTTGAT
*Il-1β*	GTCACAAGAAACCATGGCACAT	GCCCATCAGAGGCAAGGA
*Il-6*	CTGCAAGAGACTTCCATCCAGTT	AGGGAAGGCCGTGGTTGT
*Tnf- α*	GGCTGCCCCGACTACGT	ACTTTCTCCTGGTATGAGATAGCAAAT
*Hmg-coa*	GGCCCAGTGGTGCGTCTTCC	TGGTCCCACCACCCACGGTT
*Ldlr*	ACTCATGCAGCAGGAACC	GTCATTTTCACAGTCTAC
*Gapdh*	CATGGCCTTCCGTGTTCCTA	GCGGCACGTCAGATCCA

Abbreviation: *Acox1*, Peroxisomal acyl-coenzyme A oxidase 1; *Cpt1α*, Carnitine palmitoyl transferase I alpha; *Pparα*, Peroxisome proliferator-activated receptor alpha; *Atgl*, Adipose triglyceride lipase; *Hsl*, Hormone-sensitive lipase; *Mgl*, Monoacylglycerol lipase; *Acc*, Acetyl-CoA carboxylase; *Fas*, Fatty acid synthase; *Scd1*, Stearoyl-CoA 9-desaturase; *Dgat1*, Diacylglycerol O-acyltransferase; *Pgc1α*, Peroxisome proliferator-activated receptor gamma coactivator 1 alpha; *Srebp1c*, Sterol regulatory element-binding protein-1c; *Il*, Interleukin; *Tnf-α*, Tumor necrosis factor alpha; *Hmg-coa*, 3-Hydroxy-3-Methylglutaryl-Coenzyme A; *Ldlr*, Low-Density Lipoprotein Receptor; *Gapdh*, Glyceraldehyde-3-phosphate dehydrogenase.

**Table 3:** Antibodies used for Western blot analysis.

Type	Antibody	Dilution Factor	Corporation	Catalog Number
Primary	*p*-AMPK	1:1000	Cell Signaling	2531
	t-AMPK	1:1000	Cell Signaling	2532
	GAPDH	1:2000	Santa Cruz	365,062
Secondary	Anti-rabbit IgG	1:3000	Cell Signaling	7074
	Anti-mouse IgG	1:1000	Cell Signaling	7076

Abbreviation: *p*-AMPK, Phospho-AMP-activated protein kinase; t-AMPK, Total-AMP-activated protein kinase; GAPDH, Glyceraldehyde-3-phosphate dehydrogenase; Anti-rabbit IgG, Peroxidase antibody to rabbit Immunoglobulin G produced in goat; Anti-mouse, Peroxidase antibody to mouse Immunoglobulin G produced in goat.

## Data Availability

The datasets used in this study are available from the corresponding author upon reasonable request.

## Abbreviations

Acc	Acetyl-Coa Carboxylase
*Acox1*	Peroxisomal Acyl-Coenzyme A Oxidase 1
ALT	Alanine Aminotransferase
AMPK	Adenosine Monophosphate-Activated Protein Kinase
AST	Aspartate Aminotransferase
AUC	Area Under the Curve
*Atgl*	Adipose Triglyceride Lipase
BAs	Bile Acids
BMI	Body Mass Index
BW	Body Weight
CFU	Colony-Forming Units
Cmax	Maximum Plasma Concentration
*Cpt1α*	Carnitine Palmitoyl Transferase I Alpha
CRF	Cardiac Risk Factor
CVD	Cardiovascular Disease
*Dgat1*	Diacylglycerol O-Acyltransferase 1
EAT	Epididymal Adipose Tissue
ELISA	Enzyme-Linked Immunosorbent Assay
*Fas*	Fatty Acid Synthase
FC	Free Cholesterol
FER	Food Efficiency Ratio
*Gapdh*	Glyceraldehyde-3-Phosphate Dehydrogenase
H&E	Hematoxylin and Eosin
HDL-C	High-Density Lipoprotein Cholesterol
HFD	High Fat Diet
*Hmg-coa*	3-Hydroxy-3-Methylglutaryl-Coenzyme A
HOMA-IR	Homeostasis Model Assessment of Insulin Resistance
*Hsl*	Hormone-Sensitive Lipase
*Il-1β*	Interleukin 1 Beta
*Il-6*	Interleukin 6
*Ldlr*	Low-Density Lipoprotein Receptor
LDL-C	Low-Density Lipoprotein Cholesterol
LL	Lactobacillus delbrueckii subsp. lactis CKDB001
MASLD	Metabolic-Associated Steatotic Liver Disease
MASH	Metabolic Dysfunction-Associated Steatohepatitis
MAT	Mesenteric Adipose Tissue
*Mgl*	Monoacylglycerol Lipase
NAFLD	Non-Alcoholic Fatty Liver Disease
NAS	Non-Alcoholic Fatty Liver Disease Activity Score
NASH	Non-Alcoholic Steatohepatitis
NCD	Normal Chow Diet
OGTT	Oral Glucose Tolerance Test
OLTT	Oral Lipid Tolerance Test
PBS	Phosphate-Buffered Saline
PC	Positive Control
*Pgc1α*	Peroxisome Proliferator-Activated Receptor Gamma Coactivator 1 Alpha
PPAR-GAMMA	Peroxisome Proliferator-Activated Receptor Gamma
*Pparα*	Peroxisome Proliferator-Activated Receptor Alpha
QRT-PCR	Quantitative Reverse Transcription-Polymerase Chain Reaction
RIPA	Radioimmunoprecipitation Assay
SAT	Subcutaneous Adipose Tissue
*Scd1*	Stearoyl-CoA 9-Desaturase
SDS	Standard Deviations
*Srebp1c*	Sterol Regulatory Element-Binding Protein 1c
T2D	Type 2 Diabetes
TC	Total Cholesterol
TG	Triglycerides
TMAX	Time to Reach Maximum Plasma Concentration
*Tnf-α*	Tumor Necrosis Factor Alpha
T-AMPK	Total-AMP-Activated Protein Kinase
WAT	White Adipose Tissue
WHO	World Health Organization
